# The impact of surface chemistry on the performance of localized solar-driven evaporation system

**DOI:** 10.1038/srep13600

**Published:** 2015-09-04

**Authors:** Shengtao Yu, Yao Zhang, Haoze Duan, Yanming Liu, Xiaojun Quan, Peng Tao, Wen Shang, Jianbo Wu, Chengyi Song, Tao Deng

**Affiliations:** 1 State Key Laboratory of Metal Matrix Composites, School of Materials Science and Engineering, Shanghai Jiao Tong University, 800 Dong Chuan Road, Shanghai 200240, P.R. China; 2 MOE Key Laboratory for Power Machinery and Engineering, School of Mechanical Engineering, Shanghai Jiao Tong University, 800 Dong Chuan Road, Shanghai 200240, P.R. China

## Abstract

This report investigates the influence of surface chemistry (or wettability) on the evaporation performance of free-standing double-layered thin film on the surface of water. Such newly developed evaporation system is composed of top plasmonic light-to-heat conversion layer and bottom porous supporting layer. Under solar light illumination, the induced plasmonic heat will be localized within the film. By modulating the wettability of such evaporation system through the control of surface chemistry, the evaporation rates are differentiated between hydrophilized and hydrophobized anodic aluminum oxide membrane-based double layered thin films. Additionally, this work demonstrated that the evaporation rate mainly depends on the wettability of bottom supporting layer rather than that of top light-to-heat conversion layer. The findings in this study not only elucidate the role of surface chemistry of each layer of such double-layered evaporation system, but also provide additional design guidelines for such localized evaporation system in applications including desalination, distillation and power generation.

This paper studies the impact of surface chemistry on the evaporation performance of the localized solar-driven evaporation system using anodic aluminum oxide (AAO)-based gold nanoparticle (AuNP) films. Through surface group functionalization and modulation, the surface chemistry of the top light-to-heat conversion layer and the bottom supporting layer were tailored to study the effect (Fig. 1a). The study shows that the surface chemistry of bottom supporting layer plays a more important role in affecting the evaporation performance than that of top light-to-heat conversion layer. This work provides the systematic study of the surface chemistry in modulating the heat localized evaporation and the findings will help further improve the design of such evaporation system.

Evaporation is a critical phase change phenomenon in many industrial processes, such as in desalination, distillation, and power generation^1^,^2^,^3^. Solar driven evaporation has been considered as one of the promising solar energy harvesting technologies to utilize clean and renewable solar radiation^4^,^5^,^6^. Compared to the bulk heating of evaporation liquid, the localized heating through trapping of solar energy at the evaporative interfaces shows significantly improved evaporation performance^5^,^7^,^8^,^9^. Taking inspiration from biological system, we fabricated the solar-energy-trapping system consisting of airlaid paper (the bottom supporting layer) and the self-assembled plasmonic AuNP film (the top light-to-heat conversion layer)^9^. Plasmonic heating, induced by plasmonic nanostructures under light illumination, has opened up an emerging area of critical interest due to its wide range of applications such as photothermal therapeutics, thermo-enhanced catalysis, delivery agents, thermal storage and thermal detection^10^,^11^,^12^,^13^,^14^,^15^,^16^. In our newly developed evaporation system, plasmonic heating has been applied to convert solar energy into heat at the air/water interface and high performance of localized water vapor generation was achieved^7^,^9^. Apart from plasmonics-based light-to-heat conversion, Chen and his coworkers have recently used the light-to-heat conversion layer based on the intrinsic light absorption of graphite and reported that solar-powered water vapor can be generated efficiently by applying a double-layered structure composed of light absorbing exfoliate graphite (top light-to-heat conversion layer) and insulating carbon foam (bottom supporting layer)^5^. While both systems achieved high efficiency in evaporation^5^,^9^, the impact of surface chemistry, or more specifically, the surface wettability on the evaporation performance was not studied. In biological processes, such as natural leaf transpiration, hydrophobic surfaces of plant leaves impede the water evaporation to prevent water loss while the spreading water on the hydrophilic surfaces of plant leaves evaporates rapidly to reduce the growth of micro-organisms (Fig. 1b,c)^17^. By contrast, in the highly efficient artificial evaporation systems mentioned above, the top AuNP layer is hydrophobic in the paper based system and the top graphite layer is hydrophilic in the carbon foam based system^5^,^9^,^18^. To understand the role of the surface wettability, this work intends to study *how and to what extent the surface chemistry of both the top light-to-heat conversion layer and the bottom supporting layer in the floating double-layered geometry controls the speed of water evaporation*.

In this study, nanoporous AAO membrane was used as the bottom supporting layer to provide mechanical stability for the top AuNP film layer. AAO membrane, possessing superior transport and the tunability in selectivity, has drawn considerable attention in a wide range of chemical and biological applications, especially in chemical separation, catalysis, nanotemplating and biosensor^19^,^20^,^21^,^22^. The well-established protocols in chemically modifying AAO surfaces also enable the tailoring of surface chemistry of the supporting layer^23^. To study the impact of surface chemistry on the evaporation efficiency, we performed chemical hydrophilization or hydrophobization on both the top light-to-heat conversion layer (AuNP film) and bottom supporting substrate (AAO) of double-layered system, which is called AAO-based AuNP film (AANF) in this report (Fig. 1d). Through the modulation of surface chemistry, we hope not only to study the wettability effect but also to explore a possible approach in tuning the evaporation performance of the system. Tunable property across different application situation is one of the most important criteria to evaluate the wide applicability of functional material systems. This work demonstrates that evaporation rate could be tuned by the chemically modified AANF, which behaves like a “water gate” that controls the rate of water vapor flow. In the absence of AuNP film, water evaporates through hydrophilic AAO membranes at a similar rate as the hydrophobic ones under the same light illumination. However, in the presence of AuNP film, water evaporation rate of hydrophobic AAO membranes significantly lags behind that of hydrophilic ones. In other words, AuNP film differentiates the water evaporation rates through hydrophilic and hydrophobic AAO membranes and amplifies the difference. This difference between relative evaporation rates (i.e. the evaporation rate ratio of AANF to AAO membrane with the same wettability) can be enlarged when the intensity of incident light increases. Nevertheless, varying the wettability of top AuNP film has no noticeable effect on the evaporation rate. With the demonstrated hydrophilic and hydrophobic behavior of AANF and the added understanding of its evaporation mechanism, this study provides new insight in designing efficient localized evaporation system and also possibility to tune the evaporation performance of such systems.

## Results

**Figure 1:**
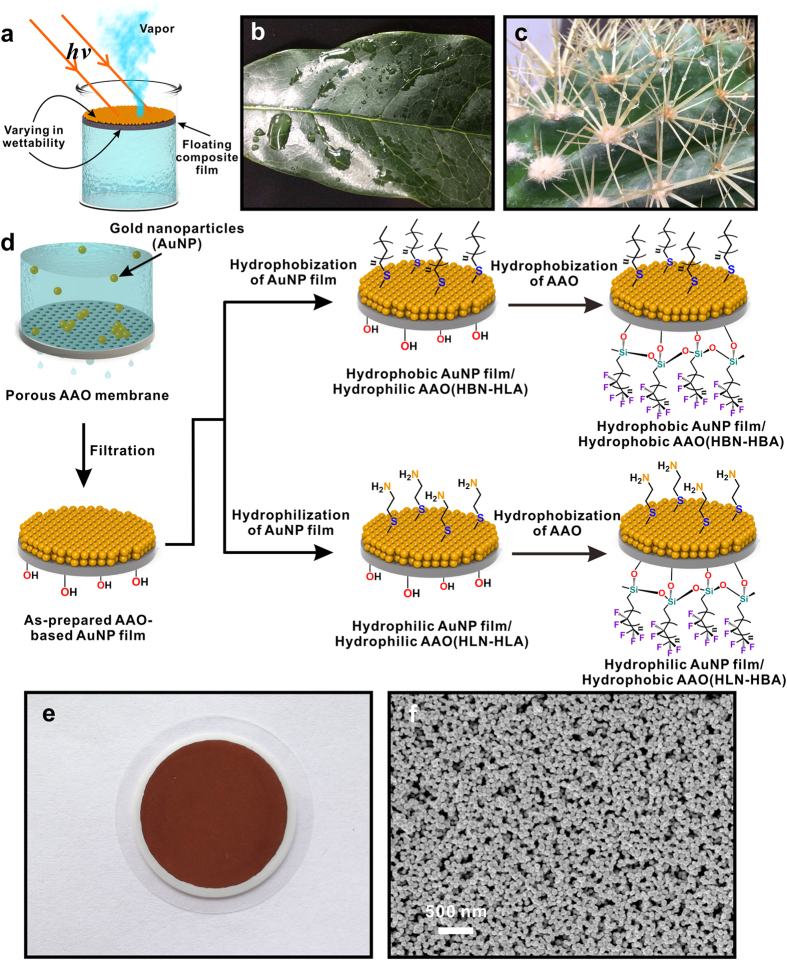
(**a**) Floating double-layered film (top: light-to-heat conversion layer; bottom: supporting layer) with varying wettability; Optical images of hydrophilic leaf of *Osmanthus fragrans* (**b**) and hydrophobic leaf of cactus (**c**); (**d**) Schematic of preparation of AAO-based AuNP film (AANF); (**e**) Optical image of AANF; (**f**) Front view SEM image of AANF. ((**a,d**) were drawn by Chengyi Song. The images in (**b,c**) were taken by Chengyi Song).

The fabrication process of AANF is schematically illustrated in Fig. 1d. After AuNPs (diameter = 104.6 ± 10.1 nm) were synthesized by seed-mediated method (Figure S1)^24^, a specific amount of concentrated aqueous AuNP solution (Volume: 3 ml; Concentration: 1.55 × 10^11^/ml) was filtered by a nanoporous AAO membrane (Whatman®6809-6002 Anodisc: pores of 200-nm diameter on bottom surface and pores of 20-nm diameter on top surface) through a vacuum filtration process and a shiny dark-red AuNP film was generated (Fig. 1e). The fresh made AANF was dried in oven overnight and stored for further chemical modification. To explore the evaporation performance of AANF with different surface wettability, we designed and fabricated the following series: 1) hydrophilic AAO membrane (HLA) alone; 2) hydrophobic AAO membrane (HBA) alone; 3) hydrophilic AuNP film sitting on hydrophilic AAO membrane (HLN-HLA); 4) hydrophobic AuNP film sitting on hydrophilic AAO membrane (HBN-HLA); 5) hydrophilic AuNP film sitting on hydrophobic AAO membrane (HLN-HBA); 6) hydrophobic AuNP film sitting on hydrophobic AAO membrane (HBN-HBA). The hydrophilic and hydrophobic treatment was simple and straightforward. AAO membrane intrinsically has many hydroxyl groups on the surface that make it hydrophilic. As shown in Fig. 1d, to make it hydrophobic, we placed AANF in a desiccator filled with 1H, 1H, 2H, 2H -perfluorooctyltri-chlorosilane (97%, Alfa Aesar) vapor under a negative pressure for about 20 minutes to make the surface of AAO membrane hydrophobic. To modify the AuNP film with controlled hydrophobicity, AANF was incubated in the mixture of alkyl thiol and acetone solution with a volume ratio V(thiol):V(acetone) = 1:49 for 3 hours. The hydrophilic surface of AuNP film was achieved by incubating AANF in the presence of 2% wt N-acetyl-L-cysteine acetone solution. The scanning electron microscopy (SEM) image shows the two-dimensional arrangement of AuNPs within the metallic film (Fig. 1f).

**Figure 2:**
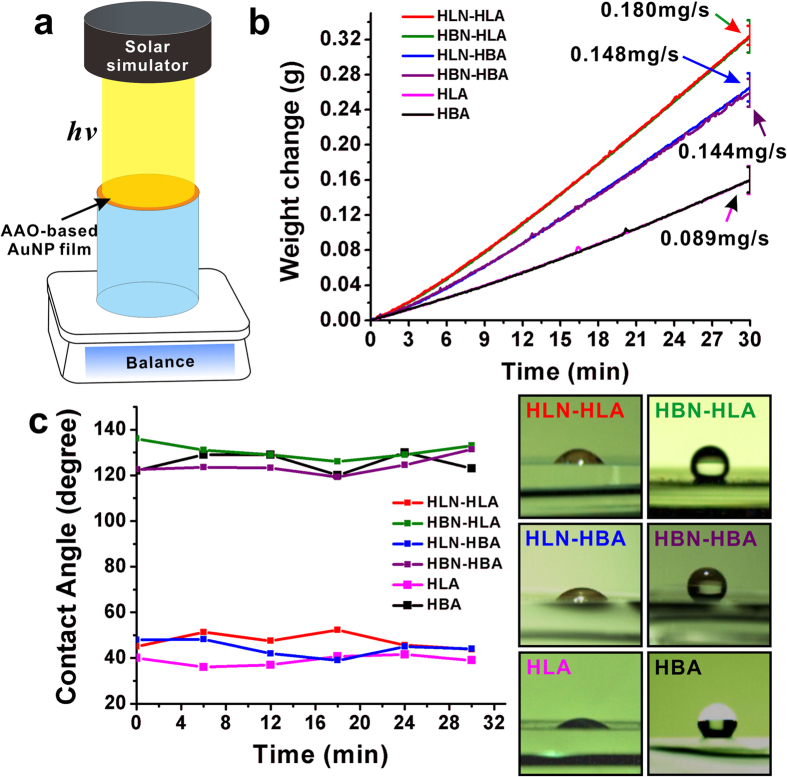
(**a**) Schematic of experimental setup of evaporation experiment; (**b**) Evaporation weight change of HLN-HLA, HBN-HLA, HLN-HBA, HBN-HBA, HLA and HBA as a function of time under Xenon lamp with power density of ~3.2 kW/m^2^; (**c**) Contact angle measurements of HLN-HLA, HBN-HLA, HLN-HBA, HBN-HBA, HLA and HBA during water evaporation process. Right side shows the optical images of water droplets on different surfaces. ((**a**) was drawn by Chengyi Song).

Figure 2a illustrates the experimental setup of evaporation performance. A 10-mL beaker full of water (NANOpure, Millipore Water Purification System; 18.2 MΩ) was placed on a 4 decimal electronic precision analytical balance (FR124CN, Ohaus Instrument, Shanghai), which can record the weight change of evaporation system at intervals of 5 seconds. A piece of AANF, with the similar diameter of the beaker, was floating on the surface of water. When a Xenon lamp (JYANG HID 922, Ju Jing Yang Electronics Co., Ltd.) with the power density of ~3.2 kW/m^2^ was set up to illuminate vertically upon the floating AANF, the generated heat would be localized within AANF^7^,^9^. To obtain the evaporation rate of AANF with different surface chemistry modification, we carefully examined the weight change of water and listed the following observations on the basis of Fig. 2b: 1) the use of either HLA or HBA resulted in almost the same water evaporation speed; 2) all the AANFs achieved higher evaporation rates than HLA or HBA alone due to the induced plasmonic heating; 3) varying wettability of AuNP film does not affect the evaporation rate of AANFs; 4) when HLA and HBA were coupled with AuNP film, the difference in evaporation rate between HLA and HBA emerges (i.e. the evaporation rate of HLN-HLA or HBN-HLA exhibits the highest evaporation rate (0.180 mg/s), which is ~1.23 times of that of HLN-HBA or HBN-HBA). To further verify the role of wettability during evaporation, we also carried out measurements of surface contact angle (Fig. 2c). A high-speed camera (X-Stream XS-4, IDT, US) recorded the process of dropping 2-μL water droplet on the AuNP film by a pipette. The contact angles were obtained through the analysis of the recorded optical images before, during and after the evaporation processes. Figure 2c shows that the contact angles of all six samples were relatively constant, implying that chemical modification was stable during water evaporation.

**Figure 3:**
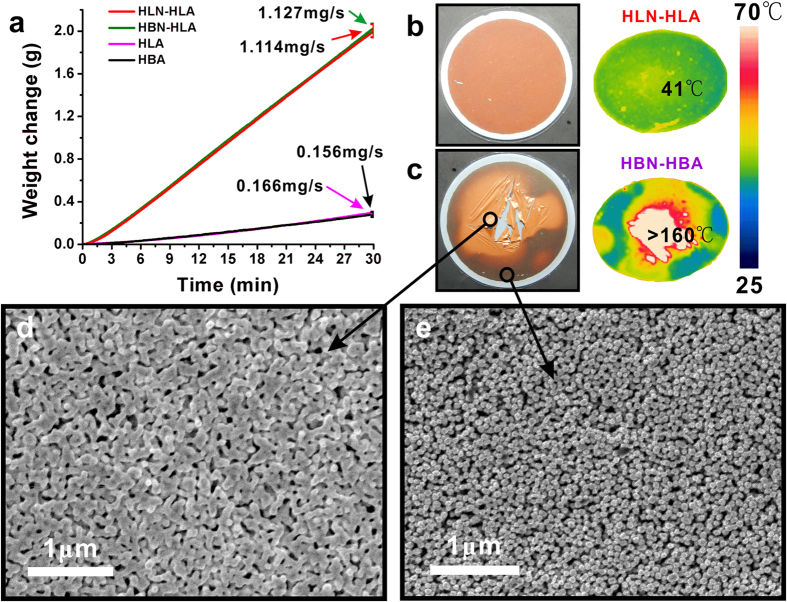
(**a**) Evaporation weight change of HLN-HLA, HBN-HLA, HLA and HBA as a function of time under Xenon lamp with power density of ~14.3 kW/m^2^; optical images and IR images of HLN-HLA (**b**) and broken HBN-HBA (**c**); (**d**,**e**) SEM images of HBN-HBA after exposed to Xenon lamp with power density of ~14.3 kW/m^2^.

Further investigation on AANF’s evaporation performance under strong light intensity was carried out by employing a Fresnel lens (Shenzhen Salens Technology Co., Ltd) to focus the Xenon light upon the surface of AANF (light power density is ~14.3 kW/m^2^), while other parameters of the setup were kept the same. As shown in Fig. 3a, the evaporation rate reached as fast as ~1.1 mg/s, which was about 6 times higher than their performance under weak light.

For hydrophobic AAO-based AANFs, however, we observed that the film quickly turned into golden shiny at the center spot and was ruptured after ~12-minute illumination (Fig. 3c), which also showed a much higher surface temperature than that of the hydrophilic AAO-based AANFs (Fig. 3b). Close examination of SEM (Quanta 250 from FEI, 20 kV) reveals that the AuNPs in the shiny ruptured area were fused together while those at the normal brown part remained as discrete particles. According to the report by Peng *et al*., the fusing temperature of AuNPs should reach to >200 °C^25^, which indicates that the local temperature of central ruptured AuNP film could be much higher than 160 °C observed in Fig. 3c due to the intensively localized plasmonic heating^26^. The fusing of AuNPs further suppressed the vapor escaping rate within the system and eventually led to the pressure build up and the rupture of the film. Though illuminated from the same focused light for 30 minutes, the hydrophilic AAO-based AANFs kept their shape and no rupture was observed.

**Figure 4:**
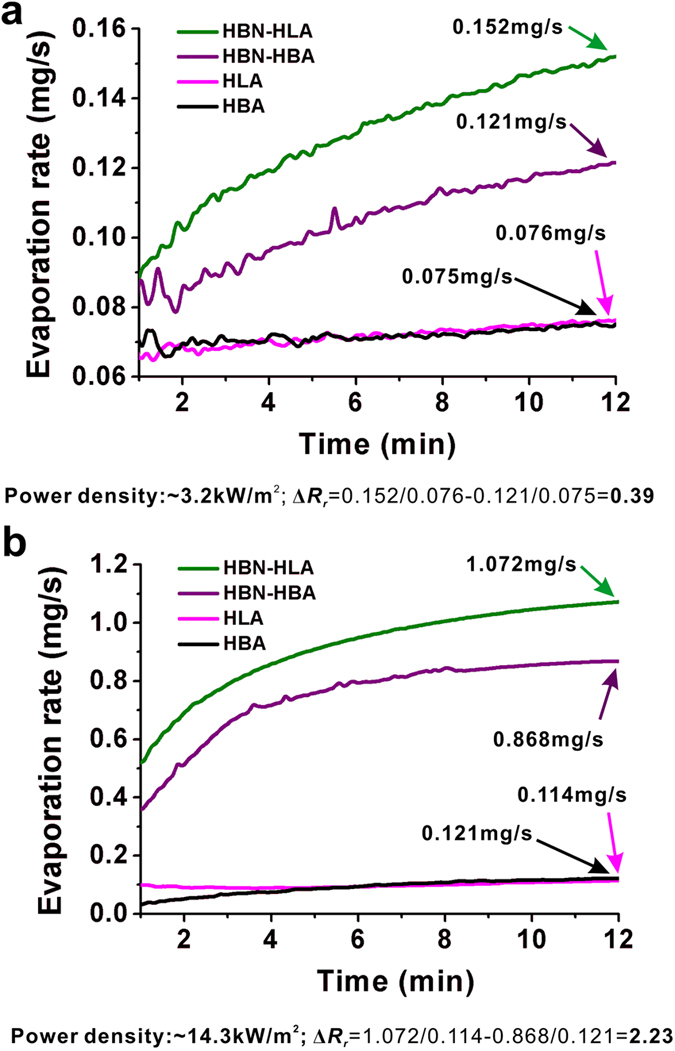
Evaporation rates of HBN-HLA and HBN-HBA illuminated under different power density of ~3.2 kW/m^2^ (a) and ~14.3 kW/m^2^ (b). The calculations of the difference between relative evaporation rate (Δ***R***_***r***_) are shown under the plots.

In order to evaluate the evaporation performance of AANFs, we calculate the difference in relative evaporation rate (Δ***R***_***r***_) to quantify the difference in evaporation rate between hydrophilic and hydrophobic AAO-based AANFs under specific incident light intensity (Equation 1 and 2):


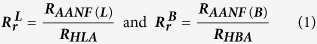



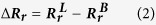


where ***R***_***AANF***(***L***)_ and ***R***_***AANF***(***B***)_ are the evaporation rates of hydrophilic and hydrophobic AAO-based AANFs, respectively; ***R***_***HLA***_ and ***R***_***HBA***_ are the evaporation rates of hydrophilic and hydrophobic AAO membrane, respectively; 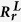 and 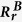 are the relative evaporation rate ratio of hydrophilic AAO-based AANF to hydrophilic AAO and hydrophobic AAO-based AANF to hydrophobic AAO, respectively; Δ***R***_***r***_ is the difference between relative evaporation rates. The calculated Δ***R***_***r***_ under different incident light intensity is shown in Fig. 4 (Δ***R***_***r***_ = 0.39, intensity = ~3.2 kW/m^2^; Δ***R***_***r***_ = 2.23, intensity = ~14.3 kW/m^2^), which shows that plasmonic nanoparticle film can differentiate the evaporation rates of hydrophilic and hydrophobic AAO during solar light driven evaporation and the difference can be amplified when the incident light intensity increases. Due to the relatively large fluctuation in evaporation during the first minute as the evaporation stabilizes, only evaporation rates between 1 and 12 minutes are shown in Fig. 4.

**Figure 5:**
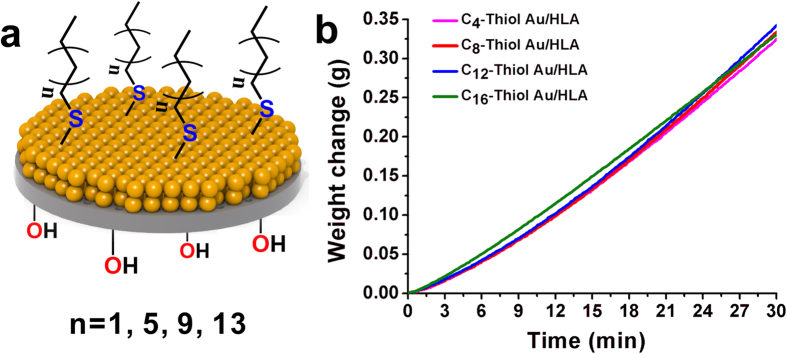
(**a**) Schematic of AuNP films sitting on the AAO membrane modified with different alkyl chains (C_4_, C_8_, C_12_, C_16_); (**b**) Evaporation weight change of AANF as a function of time. ((**a**) was drawn by Chengyi Song).

With the flexibility of controlling the chain length of alkyl thiols, we also tailored the organic tether group attached to the AuNP film surface by using thiols with different carbon chain lengths and studied the evaporation performance of HBN-HLA. Different thiols could potentially change the optical absorption performance of the AuNPs and resulted in the difference in heat localization and evaporation^27^. Thiols with 4, 8, 12, 16 carbon atoms in the straight aliphatic chain were attached to the top AuNP layer using the same experimental procedure. Figure 5 shows that the evaporation performances of these systems are almost the same, indicating the alkyl chain length does not affect evaporation rate.

## Discussion

**Figure 6:**
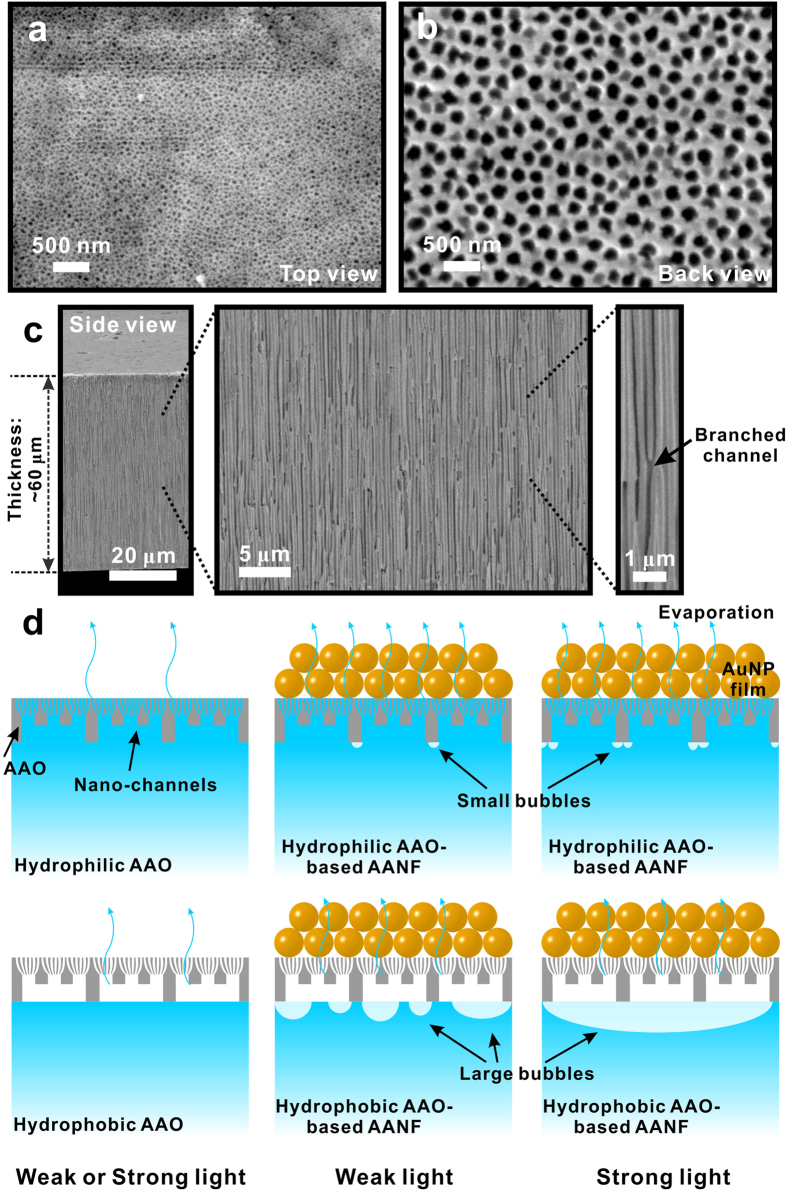
SEM images of the AAO membrane from (a) top view, (b) back view and (c) side view with different magnification. (**d**) Schematics of evaporation process of HLA, HBA and AANFs under weak (~3.2 kW/m^2^) or strong (~14.3 kW/m^2^) light illumination (not drawn to scale). ((**d**) was drawn by Chengyi Song).

To help understand the AANF evaporation performance, we further examined the morphologies of nano-porous AAO membranes by SEM and proposed an evaporation mechanism of AAO-based film in Fig. 6. Figure 6a,b show top and back view of AAO membrane, respectively. A typical cross-sectional SEM view (Fig. 6c) of the AAO membrane shows the non-intercrossing, cylindrical branched nano-channels of AAO membrane. Along the direction perpendicular to the surface, there are small parallel branches that connect the large and small nano-channels. As shown in Fig. 6d, AAOs are placed on the surface of water during evaporation process. Capillary force leverages water from bottom to the top of nano-channels within HLA while water remains at the bottom of HBA due to the water repelling nature of the hydrophobic surface. The top evaporation areas of HLA and HBA, however, are almost the same, which is consistent with our observations that HLA and HBA exhibited similar evaporation rate during the light-driven evaporation process (Figs 2b and 3a).

The evaporation performance of AAO membranes covered with AuNP films, however, was different. When AANF is exposed to simulated solar light radiation, the surface temperature of AuNP film will be increased due to the highly efficient light-to-heat conversion^28^. Such increase in temperature accelerates the rate of water evaporation. The rapid water replenishment from the bottom of AAO to the top helps cool down the heated thin film in the case with hydrophilic AAO (Fig. 3b). For hydrophobic AAO, the surface temperature of AuNP film will continuously increase and the volume of accumulated hot water vapor will substantially expand. Eventually the pressure exceeds the mechanical tolerance of AuNP film, and it will lead to rupture, which was observed in hydrophobic AAO-based AANF under strong light illumination (Fig. 3c).

Interestingly, during water evaporation, we also observed the periodic bubble formation, growth and departure process under hydrophobic AAO-based AANF’s bottom surface and the cycle length of such process would vary when the incident light intensity changed. Although small bubbles also formed under the hydrophilic AAO-based AANFs, we did not observe periodic bubble departure during the whole process of evaporation. As shown in Figure S2, under weak light (~3.2 kW/m^2^) illumination, small bubbles formed and merged under hydrophobic AAO-based AANFs and the cycle of bubble formation, growth and departure were in the time scale of several minutes. In contrast with weak light, under strong light (~14.3 kW/m^2^) illumination, huge bubbles (~one or two centimeters in diameter) formed and the cycle length of bubble formation, growth and departure was reduced to the time scale of ~10 s of seconds. These bubbles formed under hydrophobic AAO-based AANFs can act as insulating layer, which might leads to smaller temperature rise at the water surface and lower evaporation rate than those of hydrophilic AAO-based AANFs (Fig. 4).

In this work, we investigated the plasmonic heating induced evaporation performance by tailoring the wettability of both the top light-to-heat conversion layer and the bottom supporting layer. Through systematic variation of the surface chemistry and different combination of surface wettability of the AuNP films supported by AAO membranes, both the evaporation performance and the top surface wettability were recorded during the evaporation process under both weak and strong light illumination. The wettability of the top surfaces did not play a key role in controlling the evaporation performance, which clarified the reported performances of localized water vapor generation systems with hydrophilic top^5^ or hydrophobic top^7^,^9^. The wettability of the bottom surfaces, however, controlled the performance of such localized steam generation systems: hydrophilic supporting layers resulted in better and more stable performance than the hydrophobic supporting layers. The bubbles formed under hydrophobic AAO membrane acted as an insulting layer that limited both the heat and mass transport, which resulted in the decreased evaporation performance of the system. The use of hydrophobic supporting layers led to the undesired heat accumulation and even generated film rupture under strong light illumination. The findings in this work provide new insight of roles played by the surface chemistry for the localized water vapor generation system, which will help expand the design guideline for the use of such systems in power generation, autoclave, and desalination.

## Methods

### Materials and Instrumentations

All chemicals were obtained from commercial sources and used without further purification. UV-Vis Spectra were collected on UV-Vis spectrometer (Ocean Optics, Model: HR2000 + CG). The IR camera was calibrated using a calibrated thermocouple (Model: K-H-GGF; Beijing Qiaomu Automation Technology Company; uncertainty quoted from manufacturer: ~0.2 °C) with the heating of AANF floating on the surface of water using an electronic heater. The measurement uncertainty of the calibrated IR camera is ~1.0 °C in the temperature range we studied.

### Preparation of colloidal metallic nanoparticles

#### Synthesis of 10-nm gold nanoparticle seeds

Solution of 38.8 mM trisodium citrate dihydrate (99% Aladin) was made by directly dissolving sodium citrate powder in DI water (NANOpure, Millipore Water Purification System; 18.2 MΩ). Aqueous solution of 1 mM tetrachloroauric acid (HAuCl_4_) was prepared by dissolving tetrachloroauric acid (49 ~ 50% Au basis; Aladin) in water. 50 ml of the HAuCl_4_ aqueous solution was boiled in a 100-ml round-bottom flask with a condenser attached and the solution was intensively stirred during heating. After adding 2.5 ml of 38.8 mM sodium citrate to the boiling solution, the mixture was boiled for 15 minutes and stirred for additional 1 hour until the solution cooled down.

#### Synthesis of 100-nm colloidal gold nanoparticles

100-nm (104.6 ± 10.1 nm) AuNPs were synthesized by stepwise NH_2_OH-seeding method^24^. In the synthesis, 10-nm colloidal AuNPs were used as seeds and stock aqueous solutions of 1% HAuCl_4_ and 0.294 M NH_2_OH were used as gold source and reducing reagent, respectively.

#### Synthesis of concentrated 100-nm colloidal gold nanoparticles

The as-prepared 100-nm colloidal gold nanoparticle solution was placed in an undisturbed container to allow for the nature precipitation. The 100-nm particles precipitated at the bottom after two weeks. We collected the precipitates and re-dispersed them in DI water. Concentrated colloidal gold nanoparticle solutions were stored at room temperature for future use. All the experiments in this report used such concentrated 100-nm colloidal AuNP solutions.

## Additional Information

**How to cite this article**: Yu, S. *et al*. The impact of surface chemistry on the performance of localized solar-driven evaporation system. *Sci. Rep*. **5**, 13600; doi: 10.1038/srep13600 (2015).

## Supplementary Material

Supplementary Information
