# Supplementary material for: The impact of surface chemistry on the performance of localized solar-driven evaporation system
Source: Sci Rep. 2015 Sep 4;5:13600. doi: 10.1038/srep13600 (PMC4559801; doi:10.1038/srep13600)
Supplement: Supplementary Information [file srep13600-s1.pdf]

Supporting information for:

The impact of surface chemistry on the performance of localized solar-driven evaporation system

Shengtao Yu,[‡,1] *Yao Zhang,*[‡,1] *Haoze Duan,*[1] *Yanming Liu,*[1] *Xiaojun Quan,*[2] *Peng Tao,*[1] *Wen Shang,*[1] *Jianbo Wu,*[1] *Chengyi Song**[1] *and Tao Deng**[1]

[1]State Key Laboratory of Metal Matrix Composites, School of Materials Science and Engineering, Shanghai Jiao Tong University, 800 Dong Chuan Road, Shanghai 200240, P.R.China.

[2]MOE Key Laboratory for Power Machinery and Engineering, School of Mechanical Engineering, Shanghai Jiao Tong University, 800 Dong Chuan Road, Shanghai 200240, P.R.China.

Corresponding author: chengyi2013@sjtu.edu.cn; dengtao@sjtu.edu.cn

‡S. Yu and Y. Zhang equally contributed to this work

Supplementary Data (Figures S1-S2)

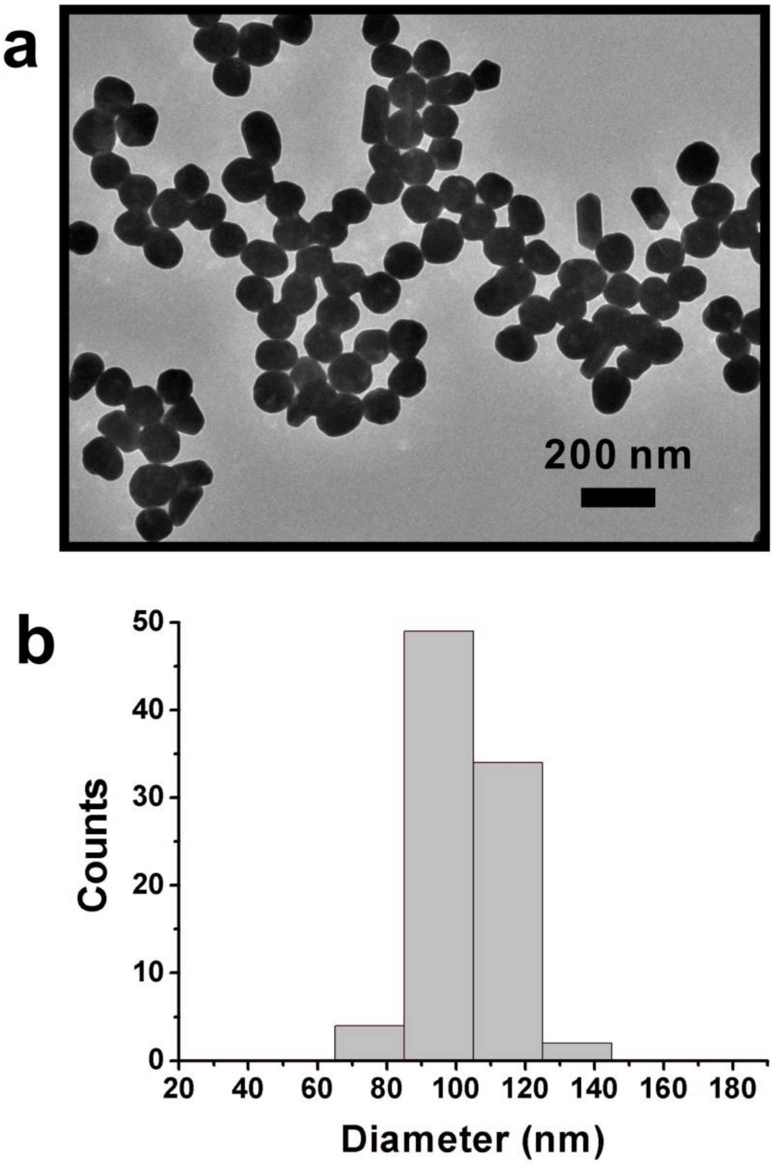

Figure S1. (a) SEM image of 100-nm AuNPs. (b) Size distribution of AuNPs (based on 89 counts; NP diameter = 104.6 $\pm$ 10.1 nm).

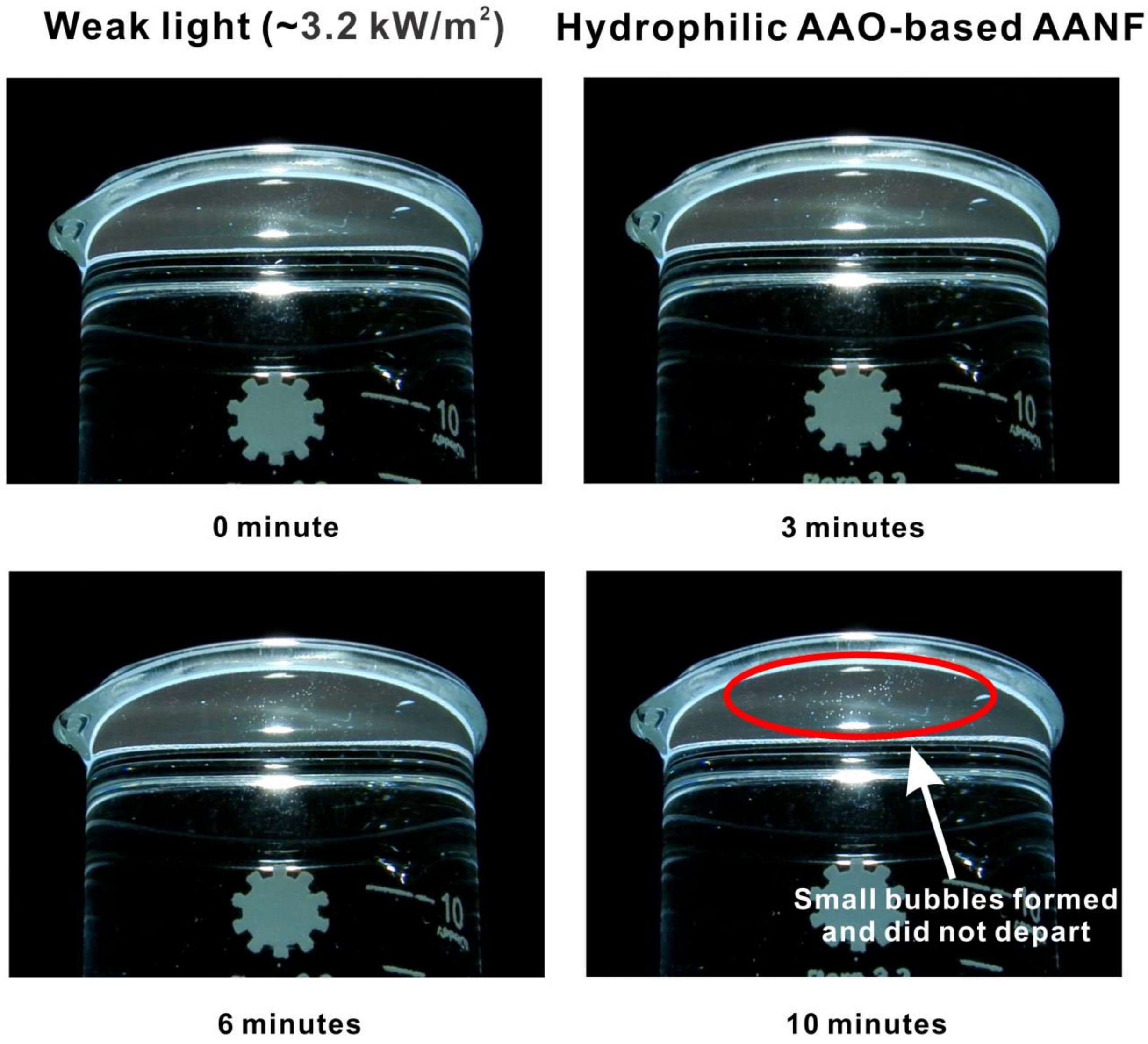

Figure S2a. Optical images of hydrophilic AAO-based AANFs exposed to weak light illumination with the power density of ~3.2 kW/m^2. A few small bubbles formed beneath hydrophilic AAO substrate after several-minute illumination. However, no bubble departure was observed during the evaporation process.

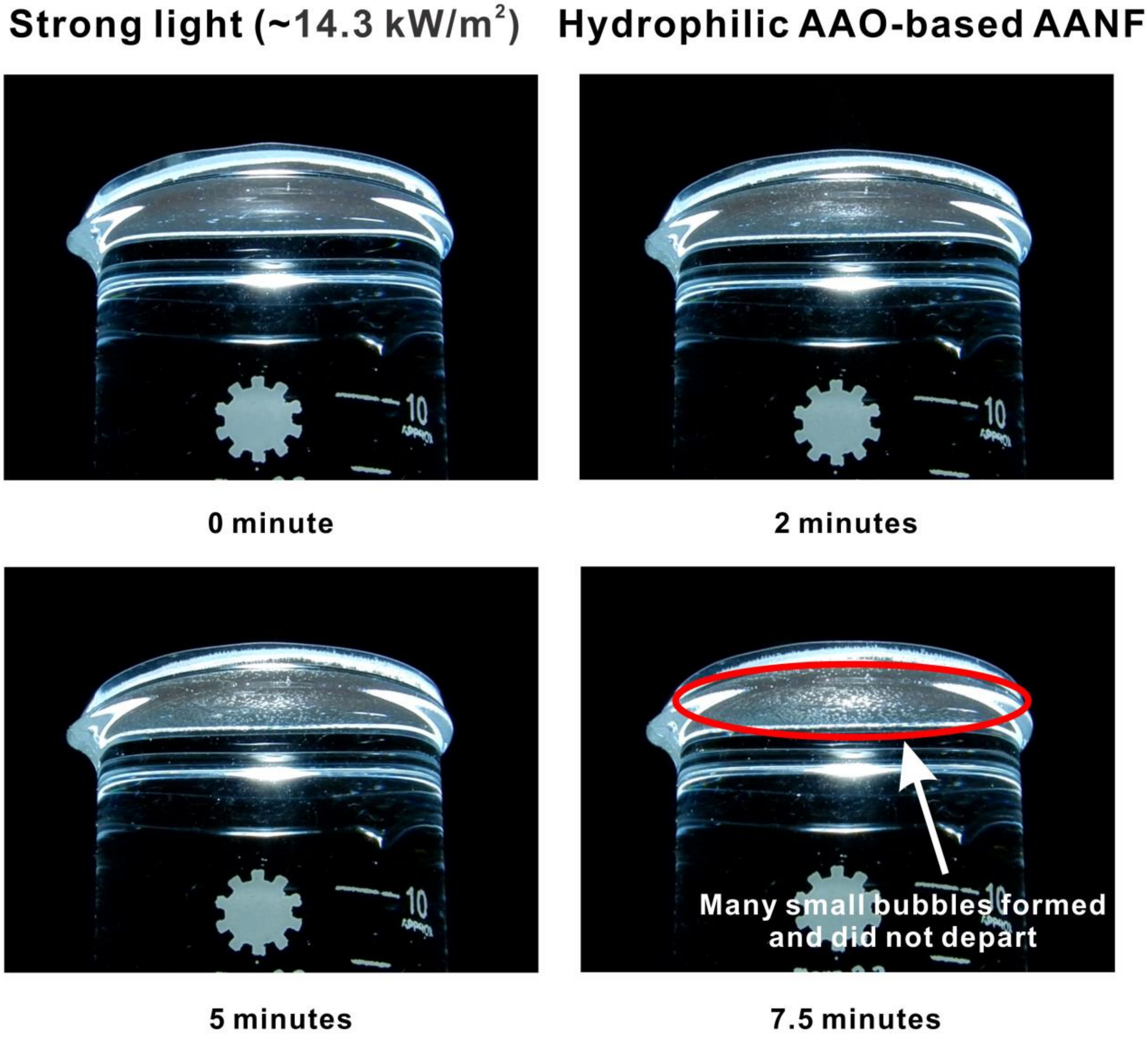

Figure S2b. Optical images of hydrophilic AAO-based AANFs exposed to strong light illumination with the power density of ~14.3 kW/m^2. Compared to the weak light illumination, more small bubbles formed beneath hydrophilic AAO substrate after several-minute illumination. However, no bubble departure was observed during the evaporation process.

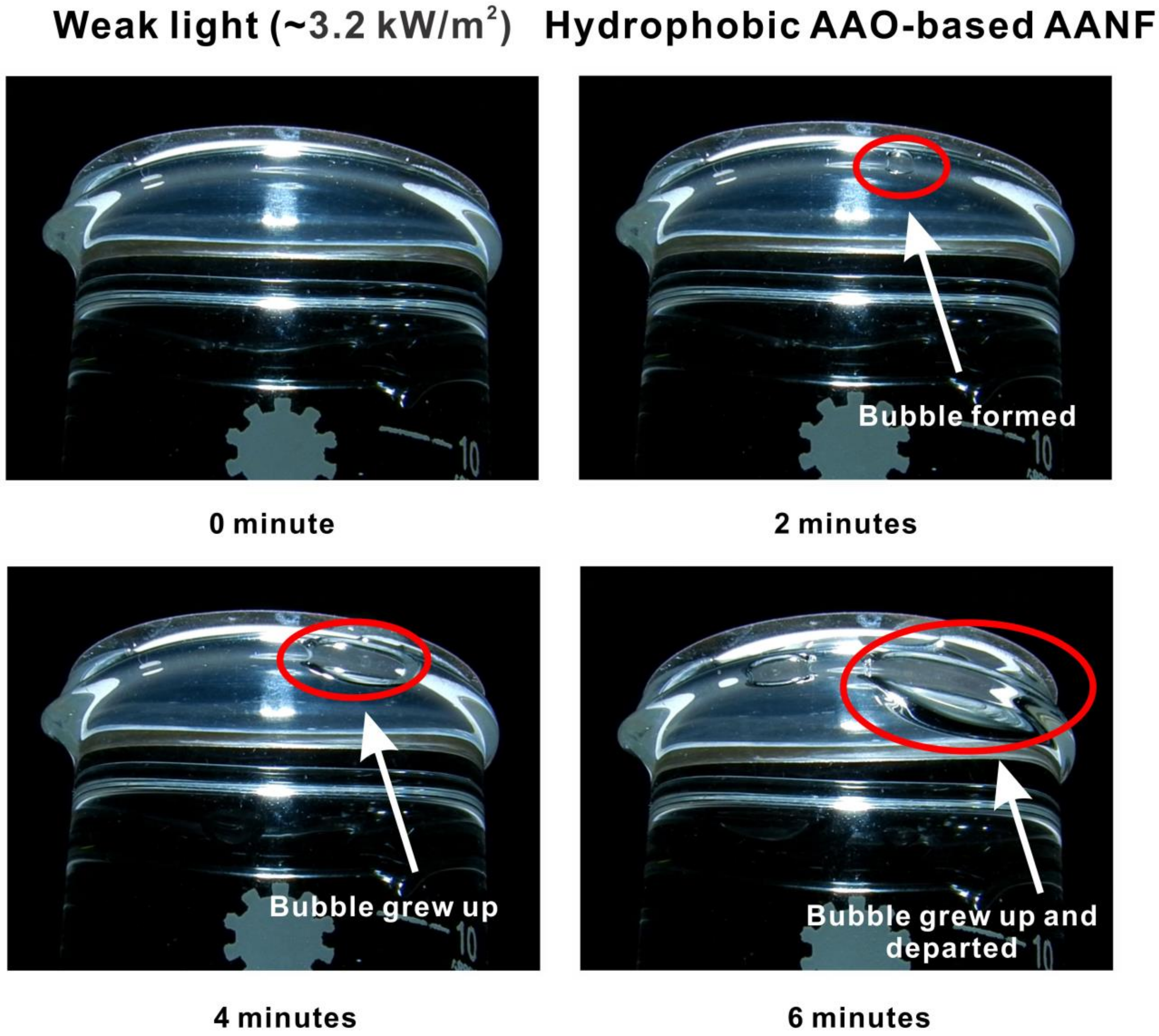

Figure S2c. Optical images of hydrophobic AAO-based AANFs exposed to weak light illumination with the power density of ~3.2 kW/m^2. Bubbles formed beneath hydrophobic AAO substrate after several-minute illumination. Bubbles gradually grew up and departed from the substrate. The cycle of bubbles formation, growth and departure is usually in the time scale of several minutes under weak light illumination.

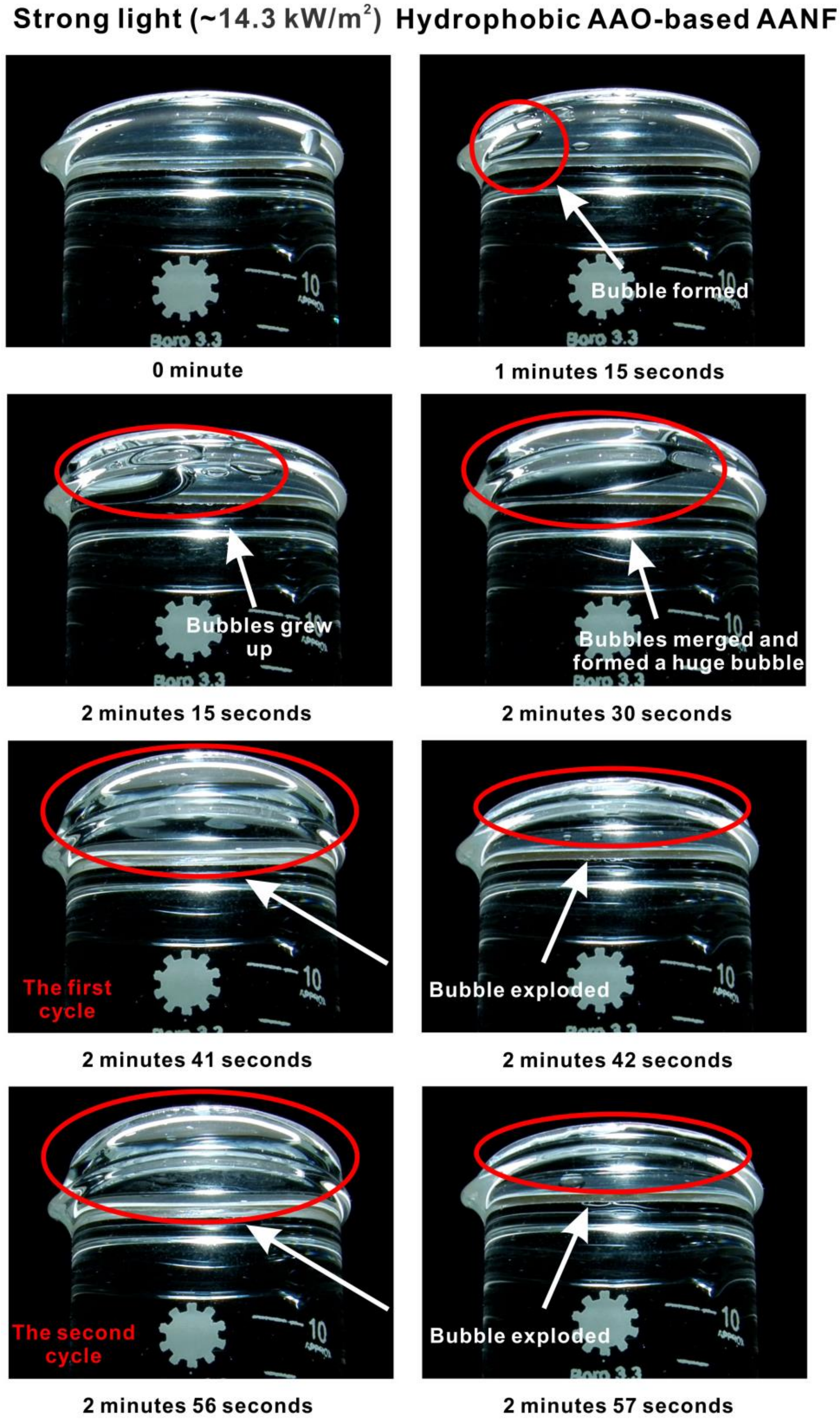

Figure S2d. Optical images of hydrophobic AAO-based AANFs exposed to strong light illumination with the power density of ~14.3 kW/m^2. Bubbles formed beneath hydrophobic AAO substrate within two-minute illumination. Bubbles progressively grew up and exploded. The

cycle of bubbles nucleation, growth and explosion is usually in the time scale of ~10s of seconds under strong light illumination.
